# Supplementary material for: Modeling glioblastoma heterogeneity as a dynamic network of cell states
Source: Mol Syst Biol. 2021 Sep 16;17(9):e10105. doi: 10.15252/msb.202010105 (PMC8444284; doi:10.15252/msb.202010105)
Supplement: Supplementary file 6 — Source Data for Figure 5 [file MSB-17-e10105-s004.zip › Figure5A_sourcedata/GSEA_3071/nosigdb_stateV.GseaPreranked.1622795159954/NB_KI_CLUSTER1.html]

Details for gene set NB\_KI\_CLUSTER1[GSEA]

|  || Dataset | state43071 |
| Phenotype | NoPhenotypeAvailable |
| Upregulated in class | na\_neg |
| GeneSet | NB\_KI\_CLUSTER1 |
| Enrichment Score (ES) | -0.7591282 |
| Normalized Enrichment Score (NES) | -3.042881 |
| Nominal p-value | 0.0 |
| FDR q-value | 0.0 |
| FWER p-Value | 0.0 |
Table: GSEA Results Summary

  

Fig 1: Enrichment plot: NB\_KI\_CLUSTER1      
 Profile of the Running ES Score & Positions of GeneSet Members on the Rank Ordered List

  

| PROBE | GENE SYMBOL | GENE\_TITLE | RANK IN GENE LIST | RANK METRIC SCORE | RUNNING ES | CORE ENRICHMENT || 1 | EEF1A1 |  |  | 3617 | -0.318 | -0.6656 | No |
| 2 | RPL13A |  |  | 4111 | -0.412 | -0.7322 | Yes |
| 3 | RPLP2 |  |  | 4156 | -0.426 | -0.7127 | Yes |
| 4 | RPS19 |  |  | 4230 | -0.449 | -0.6972 | Yes |
| 5 | RPS3A |  |  | 4266 | -0.462 | -0.6736 | Yes |
| 6 | RPL23A |  |  | 4295 | -0.471 | -0.6481 | Yes |
| 7 | RPL39 |  |  | 4323 | -0.478 | -0.6220 | Yes |
| 8 | RPL18A |  |  | 4337 | -0.483 | -0.5929 | Yes |
| 9 | RPS4X |  |  | 4344 | -0.486 | -0.5623 | Yes |
| 10 | RPS29 |  |  | 4389 | -0.498 | -0.5381 | Yes |
| 11 | RPS27 |  |  | 4435 | -0.515 | -0.5130 | Yes |
| 12 | RPL21 |  |  | 4518 | -0.548 | -0.4927 | Yes |
| 13 | RPS7 |  |  | 4574 | -0.574 | -0.4656 | Yes |
| 14 | TPT1 |  |  | 4579 | -0.576 | -0.4287 | Yes |
| 15 | RPS3 |  |  | 4632 | -0.610 | -0.3988 | Yes |
| 16 | RPL34 |  |  | 4660 | -0.624 | -0.3631 | Yes |
| 17 | RPS25 |  |  | 4663 | -0.625 | -0.3226 | Yes |
| 18 | RPL28 |  |  | 4678 | -0.632 | -0.2840 | Yes |
| 19 | RPS6 |  |  | 4751 | -0.678 | -0.2534 | Yes |
| 20 | RPL27A |  |  | 4755 | -0.680 | -0.2095 | Yes |
| 21 | RPL13 |  |  | 4787 | -0.702 | -0.1695 | Yes |
| 22 | RPS18 |  |  | 4877 | -0.784 | -0.1351 | Yes |
| 23 | RPS12 |  |  | 4888 | -0.795 | -0.0850 | Yes |
| 24 | RPL10 |  |  | 4893 | -0.803 | -0.0332 | Yes |
| 25 | RPS27A |  |  | 4909 | -0.822 | 0.0177 | Yes |
| 26 | RPS8 |  |  | 4931 | -0.847 | 0.0691 | Yes |
Table: GSEA details [plain text format]

  

Fig 2: NB\_KI\_CLUSTER1: Random ES distribution      
 Gene set null distribution of ES for **NB\_KI\_CLUSTER1**
